# *Piscine orthoreovirus* subtype 3 (PRV-3) causes heart inflammation in rainbow trout (*Oncorhynchus mykiss*)

**DOI:** 10.1186/s13567-019-0632-4

**Published:** 2019-02-18

**Authors:** Niccoló Vendramin, Dhamotharan Kannimuthu, Anne Berit Olsen, Argelia Cuenca, Lena Hammerlund Teige, Øystein Wessel, Tine Moesgaard Iburg, Maria Krudtaa Dahle, Espen Rimstad, Niels Jørgen Olesen

**Affiliations:** 1 National Institute of Aquatic Resources, Technical University of Denmark, Kongens Lyngby, Denmark; 2 Department of Food Safety and Infection Biology, Norwegian University of Life Sciences, Oslo, Norway; 3 Norwegian Veterinary Institute, Bergen, Norway; 4 Norwegian Veterinary Institute, Oslo, Norway

## Abstract

Piscine orthoreovirus (PRV) mediated diseases have emerged throughout salmonid aquaculture. Three PRV subtypes are currently reported as causative agents of or in association with diseases in different salmonid species. PRV-1 causes heart and skeletal muscle inflammation (HSMI) in Atlantic salmon (*Salmo salar*) and is associated with jaundice syndrome in farmed chinook salmon (*Oncorhynchus tshawytscha*). PRV-2 causes erythrocytic inclusion body syndrome (EIBS) in coho salmon in Japan. PRV-3 has recently been associated with a disease in rainbow trout (*Oncorhynchus mykiss*) characterized by anaemia, heart and red muscle pathology; to jaundice syndrome in coho salmon (*Oncorhynchus kisutch*). In this study, we conducted a 10-week long experimental infection trial in rainbow trout with purified PRV-3 particles to assess the causal relationship between the virus and development of heart inflammation. The monitoring the PRV-3 load in heart and spleen by RT-qPCR shows a progressive increase of viral RNA to a peak, followed by clearance without a measurable change in haematocrit. The development of characteristic cardiac histopathological findings occurred in the late phase of the trial and was associated with increased expression of CD8+, indicating cytotoxic T cell proliferation. The findings indicate that, under these experimental conditions, PRV-3 infection in rainbow trout act similarly to PRV-1 infection in Atlantic salmon with regards to immunological responses and development of heart pathology, but not in the ability to establish a persistent infection.

**Electronic supplementary material:**

The online version of this article (10.1186/s13567-019-0632-4) contains supplementary material, which is available to authorized users.

## Introduction

Piscine orthoreovirus (PRV) causes or are associated with emerging diseases in salmonid aquaculture. PRV belongs to the family *Reoviridae*, sub-family *Spinareovirinae*, genus *Orthoreovirus*. It has a double stranded RNA genome consisting of 10 segments [1]. The virion has a double protein capsid with icosahedral symmetry and no envelope [1]. PRV resists cultivation in cell culture monolayers, however, ex vivo infection of naïve red blood cells has been demonstrated [2]. Efficient propagation of the virus relies on in vivo experimental challenge in susceptible fish.

Three subtypes of PRV have been reported and are denoted as PRV-1, PRV-2, and PRV-3. Subtypes 1 and 2 have recently been identified as aetiological agents of disease in salmonids through infection with purified virus. PRV-1 causes heart and skeletal muscle inflammation (HSMI) in Atlantic salmon (*Salmo salar*) [3]. The disease was first diagnosed in 1999 in Norway [4, 5]; HSMI has also been reported in Scotland and Canada [6]. In Norway, the causative relationship between PRV-1 and HSMI was demonstrated [3]. As the disease name implies, the major histopathological findings are in located in the heart and red skeletal muscle. Affected fish show pancarditis with epicarditis, mononuclear cell infiltrations in the atrium and spongy and compact layers of the cardiac ventricle and necrotic cardiomyocytes. Severely affected fish also have red muscle inflammation [7].

PRV-2 was shown to cause erythrocytic inclusion body syndrome (EIBS) in coho salmon (*Oncorhynchus kisutch*) in 2016 [8] in Japan. The anaemic condition denoted as EIBS was first described in 1977 in rainbow trout [9] and in 1987 in chinook salmon from the Pacific Northwest of North America [10], however, as these occurrences predate the discovery of PRV-2, it remains unknown as to whether these historical EIBS cases are equivalent to the PRV-2 causing EIBS as diagnosed in Japan. To date, PRV-2 has not been reported outside of Japan.

PRV-3 was detected in 2013 following a thorough investigation of unexplained mortalities in young rainbow trout (*Oncorhynchus mykiss*) farmed in fresh water in Norway [11]. After detection of the virus, infection trials were conducted to assess its pathogenicity and the risk associated with its introduction to salmonid aquaculture in Europe. Those experimental trials showed that PRV-3 replicated in rainbow trout blood and efficiently transmitted to naïve host in a cohabitation trial, while its capacity of replicating in Atlantic salmon is limited [12]. The PRV-3 genome was sequenced and compared with PRV-1 showing that PRV-3 is more closely related to PRV-1 than PRV-2 [13].

PRV-3 antigenic properties were analyzed with antibodies raised against homologous proteins of PRV-1. Western blot analysis of PRV-3 infected blood cells have demonstrated that polyclonal rabbit antisera raised against the homologous proteins of PRV-1 cross-reacted with the PRV-3 proteins σ1, σ3, σNS, µ1, µNS, and λ1 [13].

The orthoreoviruses are, in general, ubiquitous in their respective niches. The mere detection of the virus is therefore not necessarily indicative of disease causality. For example, PRV-1 is present in almost every cohort of farmed Atlantic salmon in the marine phase [3] and PRV-3 was detected in non-diseased adult rainbow trout in Norway [14]. Recently the virus was detected in several European countries, including Scotland, Germany, Italy and Denmark both from disease outbreaks and asymptomatic fish [13], the virus might also be associated with proliferative darkening syndrome (PDS) in brown trout [15]; finally PRV-3 was detected in clinically affected coho salmon in Chile [16, 17]. The main aim of our study was to demonstrate eventual causality between PRV-3 infection in rainbow trout and development of cardiac lesions as observed in field cases and previous challenge experiments. Injection with blood from infected fish was used previously in infection trials, but did not prove that PRV-3 was in fact the sole causative agent of the disease, since other pathogens/viruses could be present in the sample.

In this study, we set out to demonstrate an eventual causality between a disease recently described in rainbow trout [11], and the associated *Piscine orthoreovirus* (PRV) [13]. To achieve this goal, PRV-3 was purified from experimentally infected rainbow trout blood and used in the challenge model previously developed to compare the infection progress and disease development with a control group injected with PRV-3 infected blood.

The viral RNA kinetics in the heart and spleen and PRV protein expression in erythrocytes were assessed, along with the development of heart pathology. In addition, the immune response was monitored through gene expression analysis and detection of PRV specific antibodies.

## Materials and methods

### Virus purification

The challenge material, consisting of purified PRV-3 particles or PRV-3 infected blood, originated from intra peritoneal (i.p.) injected fish from a PRV-3 challenge trial described earlier [13]. PRV-3 particles (isolate NOR/060214) were purified from the PRV-3 infected blood (500 µL, Ct 19.7) exactly as stated in [13]. Fractions of 0.5 mL were collected using a syringe with a 23 G needle. The density of the fractions was determined by cross referencing to the refractive index [18]. The quantity of PRV-3 in the fractions was determined using RT-qPCR. Fractions with a density corresponding to that of PRV were chosen for dialysis. The purity of the samples was inspected by transmission electron microscopy (TEM) and analyzed by Next Generation Sequencing (NGS) as described by Dhamotaran et al. [13].

### Experimental challenge

Rainbow trout were obtained from eyed eggs provided by a Danish commercial fish farm officially registered free of IPNV, IHNV, VHSV and bacterial kidney disease (BKD). After iodophor disinfection, the eggs were hatched and fish were grown in the wet laboratory facilities of the European Union Reference Laboratory for fish disease (EURL, Copenhagen, Denmark) using recirculated tap water disinfected by UV light. Before infection, the specific pathogen free (SPF) rainbow trout were moved into the high containment infection facility with fresh water at a constant temperature of 12 °C ± 1 °C. Each tank was supplied with flow-through UV disinfected water (1 full water exchange per day), furthermore one recirculating unit (EHEIM Professional 4+) was added to each tank to increase water quality and reduce water usage.

A total of 500 SPF rainbow trout of 10 g in average were kept in tanks with 5 L/h flow-through fresh water renewal using the following conditions: 12 °C ± 1 °C, L:D 12:12, stocking density below 60 kg/m^3^, and feeding of 1.5% of biomass/day. The fish were divided into three groups: Negative control; purified PRV-3 particles and positive control PRV-3 infected blood.

In order to have comparable biomass in the different groups, the negative control group of 500 Lts capacity accommodated 300 rainbow trout, the two experimental tanks accommodating fish challenged with purified PRV-3 particles and positive controls were 180 Lts accommodating 100 rainbow trout each. In all tanks the ratio between injected (shedders) and cohabitants was 50:50. To set up the cohabitation trial, shedder fish were anaesthetized by immersion in water containing benzocaine (80 mg/L water), and then i.p. injected with 0.1 ml of challenge or mock inoculum. The PRV-3 RNA load in the infected blood inoculum was Ct 26.3 per 5 µL; whereas in the purified viral particle inoculum the PRV-3 load was assessed as Ct 32.7 per 5 µL. Mock infection with blood from naïve fish (tested negative for PRV-3) diluted in L-15 medium was performed in the same manner on 50% of the negative control fish. Injected fish were marked by adipose fin clipping. Sampling took place at 2, 4, 6, 8, 10 weeks post-challenge (wpc) and included six shedders and six cohabitants in the exposed tanks, whereas 2 mock injected fish and 2 negative control cohabitants where sampled. Sampling specifics are provided in Table 1.

**Table 1:** **Design of the experimental trial**

Group	Number of fish	Trial length (weeks)	Sampling time points (weeks post-challenge-wpc)^a^	Fish sampled per time point	Samples	Analysis
Negative control	150 mock injected	10	2, 4, 6, 8, 10	2	Blood	Antibody detection
					Heart	Virus qPCR; CD4 CD8
					Spleen	Virus qPCR; IFN
					Organs	Histopathology
	150 cohabitants	10	2, 4, 6, 8, 10	2	Blood	WB—antibody detection—Hct
					Heart	Virus qPCR; CD4 CD8
					Spleen	Virus qPCR; IFN
					Organs	Histopathology
Purified PRV-3 particles	50 purified PRV-3 injected	10	2, 4, 6, 8, 10	6	Blood	Antibody detection
					Heart	Virus qPCR; CD4 CD8
					Spleen	Virus qPCR
					Organs	Histopathology
	50 cohabitants	10	2, 4, 6, 8, 10	6	Blood	WB—antibody detection—Hct
					Heart	Virus qPCR; CD4 CD8
					Spleen	Virus qPCR; IFN
					Organs	Histopathology
Positive control	50 PRV-3 infected blood injected	10	2, 4, 6, 8, 10	6	Blood	Antibody detection
					Heart	Virus qPCR; CD4 CD8
					Spleen	Virus qPCR
					Organs	Histopathology
	50 cohabitants	10	2, 4, 6, 8, 10	6	Blood	WB—antibody detection—Hct
					Heart	Virus qPCR; CD4 CD8
					Spleen	Virus qPCR; IFN
					Organs	Histopathology

^a^In addition 9 fish were collected prior to exposure and investigated for presence of PRV-3 and heart histopathology.

### Sampling

Upon sampling, fish were euthanized with benzocaine (800 mg/L). Blood was collected from the caudal vein in heparinized tubes (Kruuse ltd UK). Heparinized blood was centrifuged (130* g* for 10 min) and plasma and blood cells were separated. Blood was used for Western blot (WB) and plasma for assessing specific antibody. An aliquot of heparinized blood was centrifuged (12 000* g* for 5 min) in glass microhematocrit tubes (Vitrex Medical A/S) with specific centrifuge (Nüve) and haematocrit (hct) assessed visually with specific scale.

The heart was cut in two equal halves along the midsagittal axis; one half was stored in 10% neutral-buffered formalin for histopathological evaluation, and the other half was divided into two aliquots: one was stored in RNALater^®^ (ThermoFisher Scientific Inc, USA) for gene expression analysis (CD4 and CD8) and one was stored in RLT buffer (© QIAGEN) for quantifying viral RNA. The spleen was divided into three aliquots: one was stored in 10% neutral-buffered formalin, one was stored in RNALater^®^ (ThermoFisher Scientific Inc,) for gene expression analysis and one was stored in RLT buffer (QIAGEN) for quantifying viral RNA. Gill, liver, pancreas and pyloric caeca, distal intestine, red and white muscle, mid kidney were also collected and stored in 10% neutral-buffered formalin for histopathological evaluation.

### Quantification of PRV-3 in the heart and spleen

RT-qPCR was performed on RNA purified from fish tissue. After processing the sample with Tissue Lyzer (QIAGEN, Hilden, Germany), total RNA was isolated using QIAcube and the RNeasy Mini Kit (QIAGEN) according to the manufacturer’s recommendations.

RT-qPCR was performed with 5 µL of template using the QuantiTect Probe RT-PCR Kit (QIAGEN), primers and probes, and conditions as described elsewhere [11], with ROX as a reference dye.

All RT-qPCR analyses were performed using Agilent Mx3005P and Mx3000P qPCR-system (Agilent Technologies, Santa Clara, United States), and MxPro (v. 4.10) software, using the adaptive Baseline function. Cycle thresholds (Ct) were set manually to the same value in all the runs (dR = 380), in order to be able to compare Ct values among runs. Fish were considered virus positive at Ct levels below 35 and suspect between Ct 35–40; Ct values above 40 were negative.

### Western blotting

Purified PRV-3 virus particles or blood pellets pooled from three cohabitant fish were mixed with XT buffer and XT reducing agent (Bio-rad), heated for 5 min at 95 °C and loaded onto a 4–12% criterion XT bis–tris gel. Separated proteins were transferred onto a 0.2 µm PVDF membrane using Trans Blot Turbo Transfer system (Bio-rad) and incubated overnight at 4 °C with antiserum against PRV-1 proteins; anti-σ1 (1:1000) [19], anti-σ3 (1:500) [1], anti-µ1C (1:500) [19] and anti-actin (1:500) (Sigma). After washing 4 × 15 min the membranes were incubated with the secondary antibody horse radish peroxidase (HRP)-conjugated anti-rabbit IgG (Amersham, GE Healthcare, Buchinghamshire, UK) (1:20 000). The membranes were washed 4 × 15 min and stained with the Clarity Western ECL Substrate kit (Bio-rad). MagicMark was used as molecular weight ladder (XP Western Protein Standard, Invitrogen). Images were acquired using ChemiDoc XRS+ system and Image one software (Bio-rad).

### PRV immunoassay

The specific antibody response to PRV infection was measured as mean fluorescence intensity (MFI) in plasma samples of challenged rainbow trout using a bead based immunoassay as described earlier [20]. Briefly, plasma samples from cohabitant fish sampled from the the negative control, positive control and experimental groups were analysed for antibodies against µ1C. Beads, conjugated with and PRV-1 µ1C recombinant protein, were incubated with plasma harvested at 0, 4, 6, 8 and 10 wpc from cohabitants (n 6 per time point). The µ1C protein has 91.5% homology at the aminoacid level between PRV-1 and PRV-3 [13].

### Histopathology

Hearts from 60 fish exposed to purified PRV particles and 60 fish from the positive control group were sampled at regular intervals and examined by histopathology; in addition, 20 hearts from the negative control group were assessed. Prior to exposure 9 fish were sampled and analysed as well (Table 1).

Tissue samples collected in 10% neutral-buffered formalin were embedded in paraffin and processed into sections of 3–4 μm, stained with haematoxylin and eosin (H&E) and examined by light microscopy. Pathological findings in the heart were classified as (1) mild (one to a few lesions), (2) moderate (more extended distribution of lesions) and (3) severe (most of the heart sample affected) [21].

### Immune gene expression

In brief, tissues were homogenized in 650 µL QIAzol lysis reagent with 5 mm steel beads in a Tissue Lyser II for 5 min. Following chloroform extraction, 350 µL of aqueous phase was loaded into an automated QIAcube (Qiagen) for RNA purification. Total RNA concentration was measured using a NanoDrop ND-1000 spectrophotometer (Thermo Fisher Scientific). For gene expression analysis, cDNA was synthesized using 500 ng of total RNA using the QuantiTect Reverse Transcription kit (Qiagen) which includes a gDNA wipeout step. The qPCR was performed in duplicates with 10 ng of cDNA input in total volume of 10 µL per reaction using Maxima SYBR Green/ROX qPCR Master Mix (Fisher Scientific) and 500 nM forward and reverse primers (Table 2). The assay included 95 °C for 10 min, 40 cycles of 94 °C for 15 s and 60 °C for 30 s. The melting curve analysis confirmed the specificity of each SYBR qPCR assay, and elongation factor (EF1α) mRNA was used for normalization by the ΔΔCt method. In Figures 3C, D and 5 all the PRV-3 infected groups were compared to time-matched samples from negative control groups. At each time point, gene expression of fish exposed to purified PRV-3 particles and PRV-3 infected blood respectively were compared to the negative control at that specific time point. The fold changes in gene expression in the negative control group sampled at weeks 2, 4, 6, 8 and 10 were compared to the same group at week 0.

**Table 2:** **Primers used for immune gene analysis**

Gene	Primer sequences	Amplicon length	Reference	Gene Bank No.
Elongation Factor (EF1a)	GATCCAGAAGGAGGTCACCA TTACGTTCGACCTTCCATCC	150	[43]	AF498320.1
Mx	AGCTCAAACGCCTGATGAAG ACCCCACTGAAACACACCTG	142	[43]	NM_001171901
Viperin	ACGACCTCCAGCTCCCAAGT GTCCAGGTGGCTCTTCCTGC	173	[43]	AF076620.1
Interferon type 1	AAAACTGTTTGATGGGAATATGAAA TGTTTCAGTCTCCTCTCAGGTT	141	[43]	NM_001124531
Interferon gamma	CAAACTGAAAGTCCACTATAAGATCTCCA GGTCCAGCCTCTCCCTCAC	188	[44]	FM864345.1
CD-4	CCTGCTCATCCACAGCCTAT CTTCTCCTGGCTGTCTGACC	111	[43]	AY973030.1
CD-8 alpha	AGTCGTGCAAAGTGGGAAAG GGTTGCAATGGCATACAGTG	123	[43]	NM_001124263

### Statistical analysis

To assess the significant differences in immune gene expression between the control group and PRV-3 infected groups, the statistical analysis was performed using one way ANOVA with Dunnets multiple comparison test [22]. The correlation between viral load and immune transcripts were done by non-parametric Spearman correlation test [23].

## Results

### Characterization of purified PRV-3

**Figure 1:**
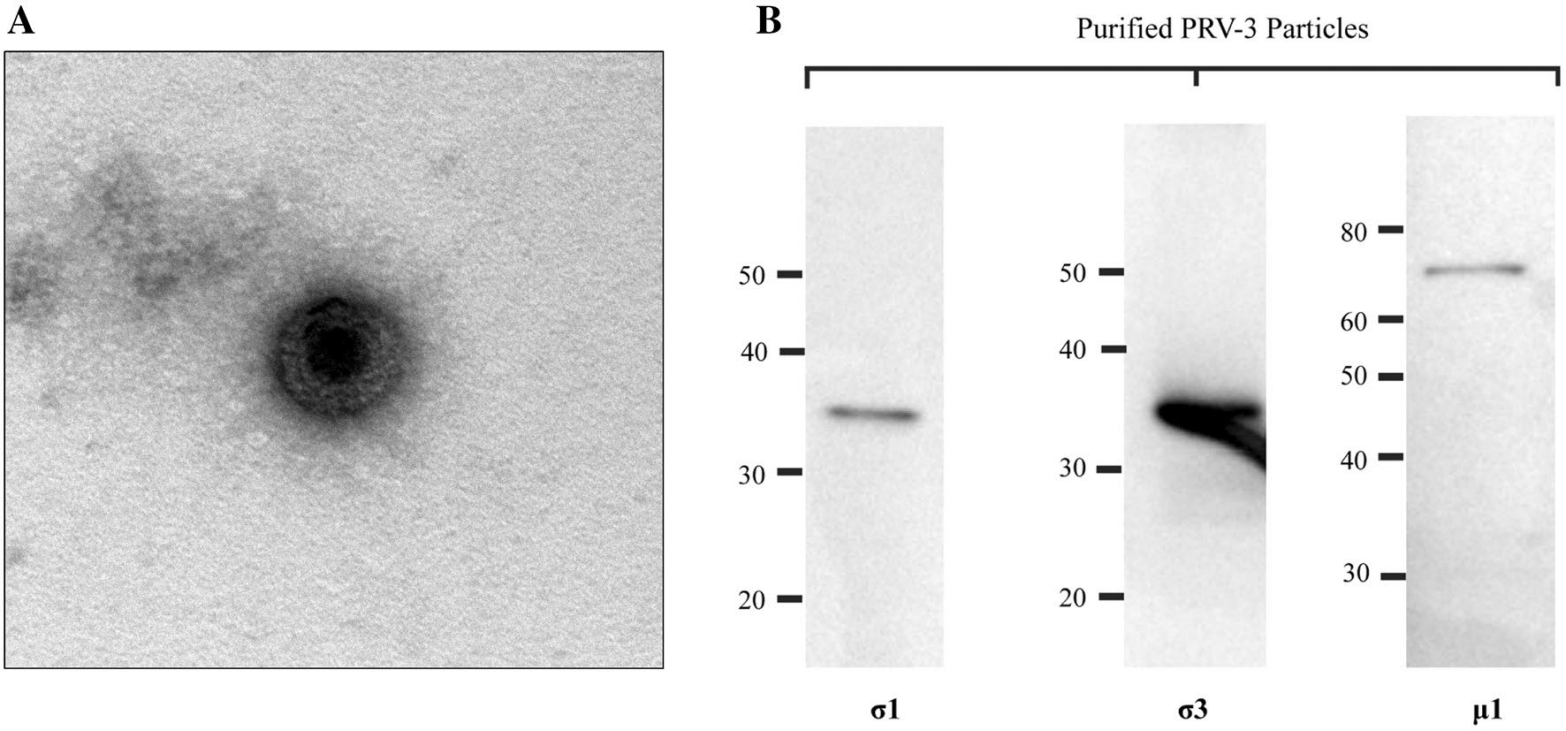
**Purified PRV-3 particles. A** Transmission electron microscopy (TEM) of purified PRV-3 viral particles. **B** Western blot analysis of proteins from purified PRV-3 particles using antiserum raised against PRV-1 σ1, σ3 and µ1 proteins. Observed bands correspond to predicted full length proteins of 35 kDa, 37 kDA and 74 kDa, respectively.

Purified PRV-3 particles were observed in TEM as spherical, non-enveloped virions of approximately 75 nm in diameter resembling PRV-1 particles [3] (Figure 1A). No other type of virus particles were found. In western blotting of purified virus, antibodies against PRV-1 σ1, σ3 and µ1 recognized the corresponding proteins of PRV-3, observed as bands of 35, 37 and 74 kDa (Figure 1B).

### PRV-3 RNA viral kinetics in heart and spleen

**Figure 2:**
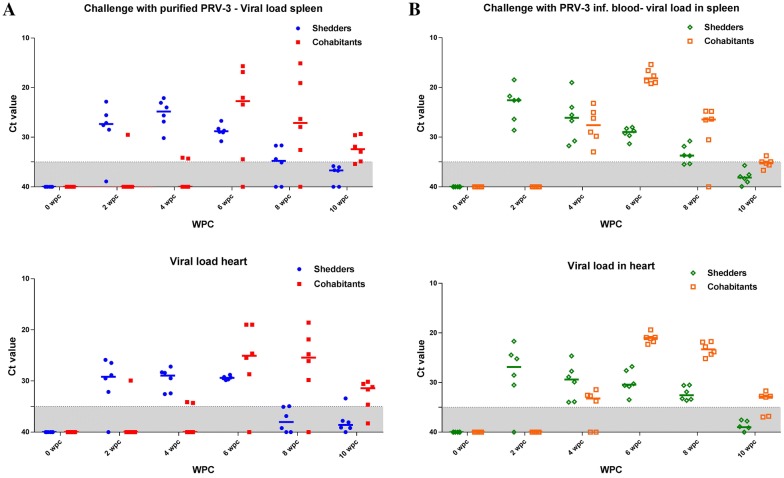
**PRV-3 infection kinetics.** RT-qPCR of PRV-3 segment S1 in spleen and heart. Colored dots indicate Ct values of individual fish while horizontal bars show the median Ct value. Samples were considered virus positive at Ct levels below 35 and suspected between Ct 35-40 (grey shaded area); Ct values above 40 are negative. **A** Shedders (Blue) and cohabitants (red) in tank challenged with purified PRV-3 particles. **B** Shedders (green) and cohabitants (orange) in tank challenged with PRV-3 infected blood.

The PRV-3 RNA load in heart and spleen showed a similar trend for the positive control group, inoculated with PRV-3 infected blood, as for the group inoculated with purified PRV-3 particles. An acute phase characterized by a peak PRV-3 load was followed by virus clearance (Figures 2A and B).

In the positive control group (injected with PRV-3 infected blood) viral RNA peaked at 2 wpc (median Ct 26.8 in the heart and 22.5 in the spleen) while the peak occurred at 6 wpc in the cohabitants (median Ct 21 in the heart and 18.1 in the spleen). In fish challenged with purified PRV-3, viral RNA peaked at 4 wpc in the injected group (median Ct 28.9 in the heart and 24.8 in the spleen) and at 6 wpc in the cohabitants (median Ct 25 in the heart and 22.7 in the spleen).

After the peak phase of infection, the number of PRV positive fish per sampling point decreased and the Ct values of the positive fish increased, indicating viral clearance. At the end of the trial (10 wpc) PRV-3 RNA could not be detected in the spleen or heart from shedders of the positive control group and only in three spleen and four heart samples from the six cohabitants. In virus-positive samples, the Ct values were close to the set detection limit (Ct 35). A similar observation was made at 10 wpc in fish infected with purified PRV-3. No PRV-3 RNA was detected in spleen samples from shedders, whereas the five positive out of six spleen samples from the cohabitants had high Ct values (> 29). Only one out of six heart samples from the shedders and five out of six from the cohabitants tested positive (Ct values > 30) at 10 wpc. No PRV-3 was detected in samples from the negative control group.

No significant differences in haematocrit were observed between the groups (data not shown).

### Histopathology

**Figure 3:**
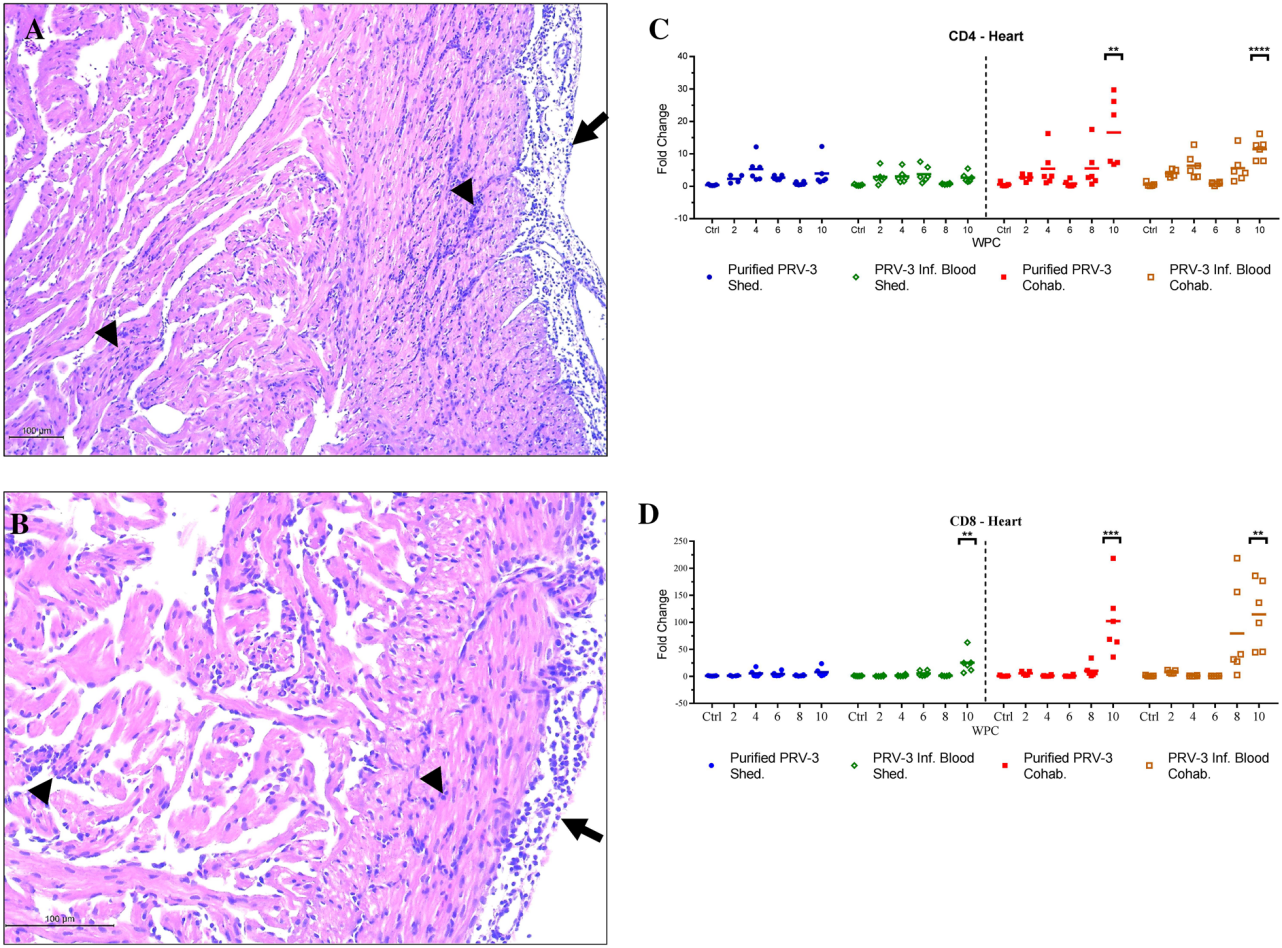
**Histopathological findings and T-cell markers in the hearts of PRV-3 infected fish. A**, **B** Rainbow trout infected with PRV-3 purified particles Epicarditis (long arrows) and inflammation in *stratum compactum* (outer layer) and *stratum spongiosum* (internal layer) of heart ventricle (arrow heads) (H&E). **C** Relative expression of the T-cell marker CD4 in the heart. The Ct values were normalized against control fish from 0 wpc. Significant differences compared to control are shown in asterisks *P < 0.05 **P < 0.001 and ***P < 0.0001. **D** Relative expression of the T-cell marker CD8 in the heart. The Ct values were normalized against control fish from 0 wpc. Significant differences compared to control are shown in asterisks *P <0.05 **P <0.001 and ***P <0.0001.

Histopathological findings in hearts consisting in the PRV-3 associated pathology described earlier [11, 12], were detected after peak load of PRV-3 in hearts in the respective groups. In the group challenged with purified PRV-3 particles, 2 shedders and 2 cohabitant fish had heart lesions, whereas 1 shedder and 7 cohabitants show heart pathology in the positive control group. Prevalence and distribution of heart lesions at each time point are shown in Table 3. The lesions observed were mild in fish infected with purified PRV-3 particles and mild to moderate in positive controls. Typical findings were epicarditis and focal to multifocal endo- and myocarditis in atrium and *stratum spongiosum* of the ventricle and perivasculitis and myocarditis in *stratum compactum* of the ventricle (Figures 3A and B). Histopathological findings consisting of epicarditis and a focal inflammatory reaction involving the interface layer between *stratum compactum* and *stratum spongiosum* were occasionally observed in all groups throughout the experiment (Additional file 1). Since they were clearly distinguishable from PRV-3 associated pathology, they were prudentially removed while assessing the development of PRV-3 pathology.

**Table 3:** **Prevalence and scores of histopathological findings in the hearts of PRV-3 challenged fish**

WPC	PRV-3 purified particles						PRV-3 infected blood					
	Shedder	Score	Ct	Cohab.	Score	Ct	Shedder	Score	Ct	Cohab.	Score	Ct
2	0/6			0/6			0/6			0/6		
4	0/6			0/6			0/6			0/6		
6	1/6	1	29.5	0/6			0/6			0/6		
8	0/6			0/6			0/6			2/6	1.5	21.8
											1.5	22.8
10	1/6	1	> 35	2/6	1	31.2	1/6	1	> 35	5/6	1	32.7
					1	30.1					1	32.7
											1	> 35
											1	31.7
											1	36.8
Positive hearts	2			2			1			7		

Proportion of positive fish per time point, histopathological score and Ct values of the positive hearts are given.

### PRV-3 protein detection

**Figure 4:**
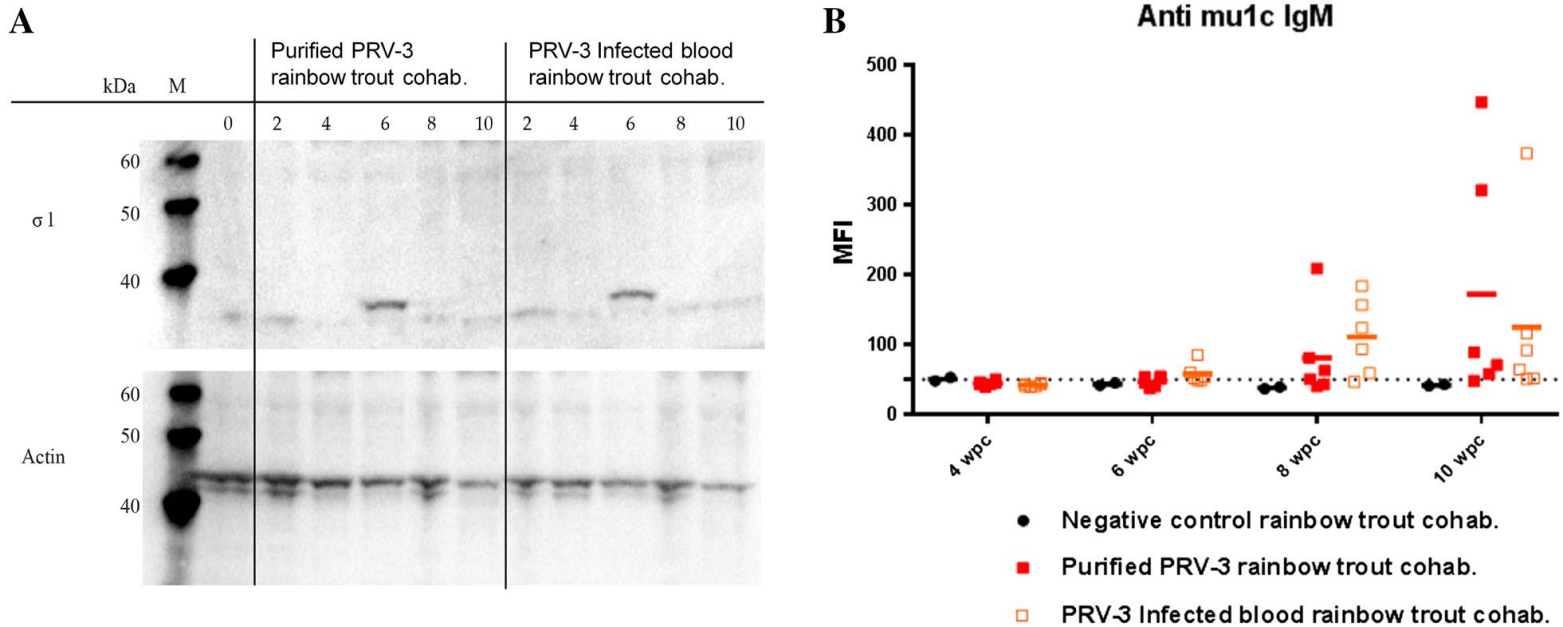
**PRV-3 proteins in blood cells and anti-PRV-3 antibodies in plasma. A** Western blot analysis of blood cell pellets from cohabitants to purified PRV-3 injected fish. **B** Multiplex analysis of PRV-3 µ1c specific antibodies in cohabitant fish measured in Mean fluorescence intensity (MFI). Six fish were tested per sampling and group.

Western blot analysis of blood cells from cohabitants of fish injected with PRV-3 purified particles or infected blood, detected σ1 viral proteins at 6 wpc, correlating with the peak phase of infection. No viral proteins were detected in blood cells after 6 wpc (Figure 4A).

### Development of specific antibodies against PRV

There was a significant increase in µ1C-specific IgM in plasma from PRV-3 infected cohabitant fish compared to plasma of uninfected fish at 8 wpc, and the production continued to increase at 10 wpc. Notably, large variations between individuals were observed (Figure 4B).

At 6 wpc the group challenged with PRV-3 infected blood had higher specific antibody levels than the purified PRV-3 particle group, but this was opposite at 10 wpc.

### Immune gene expression

**Figure 5:**
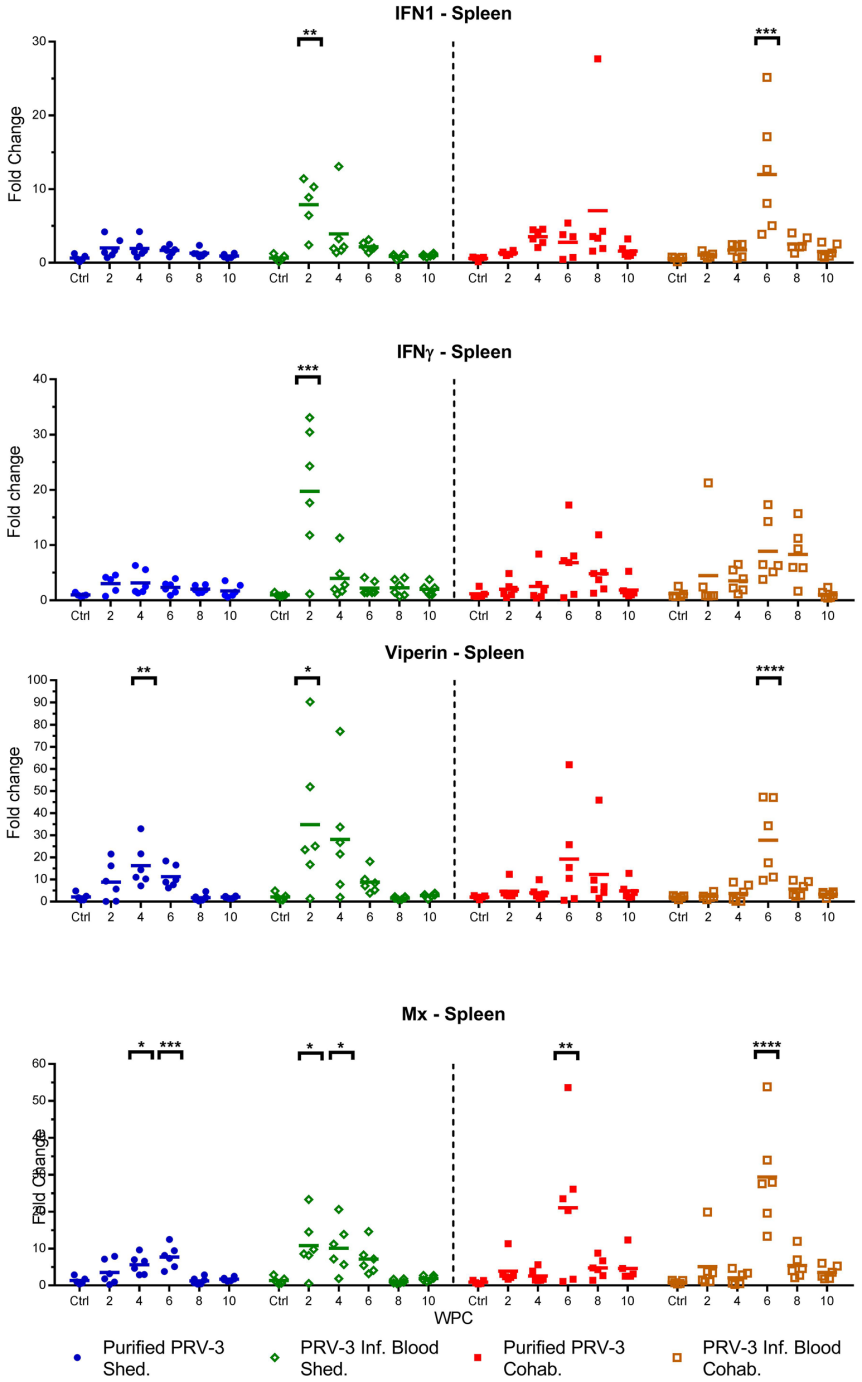
**Innate antiviral immune gene expression in spleen of PRV-3 challenged fish.** Relative expression of IFN1, IFNγ, Mx, and Viperin in controls, pure virus injected fish, blood pellet injected fish and their respective cohabitant groups (*n* = 6). The Ct values were normalized against control fish from 0 wpc. Significant differences compared to control are shown in asterisks *P <0.05 **P <0.01, ***P <0.001, ****P <0.0001.

The innate antiviral immune response following PRV-3 infection was targeted by measuring Mx, Viperin, interferon type 1 and interferon γ gene expression patterns in spleen. The T cell response in the heart was analyzed by targeting the T-cell markers CD4 and CD8. The Mx and Viperin gene expressions were significantly upregulated in the groups challenged with PRV-3 infected blood and purified virus particles, and correlated well with the viral load (Spearman r = 0.76) (Figure 5). In the shedders, the antiviral gene expression increased significantly along with the viral load at 2 wpc and 4 wpc, and subsided after 4 wpc. In the cohabitants, the antiviral gene expression shows an acute peak at 6 wpc corresponding with the peak viral load. Both viral load and antiviral gene expression decreased after 6 wpc. In both cohabitant groups, CD4 and CD8 expression in heart remained equivalent to the 0 wpc control level until 8 wpc. The CD8 gene expression shows a particularly high increase (100-fold) peaking at 10 wpc (Figures 3C and D). The CD4 expression was also significantly higher at 10 wpc. The T cell marker gene expression did not correlate with the viral peak, but with the inflammation scores in the heart.

## Discussion

The major aim of this study was to investigate an eventual association between PRV-3 infection in rainbow trout and the development of heart pathology consistent with cardiac lesions observed in natural disease outbreaks associated with PRV-3 infection. The detection of PRV-3 RNA in apparently healthy fish has raised questions in relation to the causative relationship between the virus and the disease. Koch’s postulates have historically defined the criteria to demonstrate a causative relationship between infecting agents and disease [24]. Fulfillment of Koch’s postulate relies on four main criteria, which are the presence of the disease agent in all disease cases; the possibility to isolate the agent in pure culture, the development of disease after the agent is inoculated in susceptible hosts and the re-isolation of the agent from the experimentally infected host. These criteria cannot be fulfilled for all disease-causing agents who resist culturing. According to the Rivers postulate, the viral agent should be isolated from a diseased individual and should induce the same disease when challenged with the purified viral inoculum as cell free extract and produce antibodies in a disease free individual of the same species [25]. The development of sequence based detection methods have led to further revision [26]. In recent years Atlantic salmon farming has in particular been challenged with a number of pathogens that resist cultivation [27] such as PRV-1 [3], piscine myocarditis virus (PMCV) [28, 29], and salmon gill poxvirus (SGPV) [30, 31]. The diagnostic investigations that followed the appearance of a new disease in farmed rainbow trout in Norway in 2013 [11] led to the detection of a virus with 80% gene sequence homology to PRV-1, and further characterization of the virus concluded that it was a subtype of PRV, named PRV-3. Infectious trials were conducted to assess the risk associated with introduction of the new virus in salmonid aquaculture [12], which indicated that the virus infects and replicates more effectively in rainbow trout than in Atlantic salmon. PRV-3 has been detected in several salmonid farming countries including Scotland, Germany, Italy, Denmark and Chile [13, 16, 17], but not always associated with clinical disease. Therefore, further investigations of the host–pathogen interaction could provide knowledge of benefit for fish health management.

The resistance of PRV to in vitro cultivation in available cell lines led to an approach where the virus was purified from experimentally infected fish as demonstrated by Wessel et al. [3] for PRV-1 and by Takano et al. [8] for PRV-2. Here, we adopted this approach by propagating PRV-3 in experimentally infected rainbow trout and purifying the virus by density CsCl gradient ultracentrifugation, as described by Wessel et al. [3]. The purity of viral particles was confirmed by analyzing the fraction by Next Generation Sequencing-NGS [13]. In EM, PRV-3 particles show similar features to the one displayed by purified PRV-1, i.e. the spherical particles with icosahedral symmetry with a diameter of approximately 75 nm, and two concentric electron dense layers representing the double capsid (Figure 1A).

Notably, fish exposed to PRV-3 by cohabitation had higher viral loads than the injected groups, suggesting that the cohabitation challenge model is suitable for investigation of PRV infection.

Fish challenged with purified PRV-3 by cohabitation were infected, as seen by the presence of σ1 protein at 6 wpc as demonstrated by western blot of pelleted blood cells and by the increase of qPCR positive fish after cohabitation. The timing of this is similar to PRV-1 infection in Atlantic salmon [32].

PRV-1 establishes a persistent infection in Atlantic salmon, and viral RNA can be detected for at least 57 wpc [33]. On the contrary, we observed that experimental PRV-3 infection in rainbow trout was characterized by significant clearance over time, in line with a previous study [12]. More specifically, the number of positive fish and virus levels per sampling point decreased after peak viremia. This trend of the infection kinetics was observed in both infected groups in our trial. In the positive control tank the injected fish reached peak virus levels already 2 wpc, whereas in the group injected with purified PRV-3 particles the peak was 4 wpc. Most likely, this difference can be explained by lower viral load in the initial inoculum, i.e. Ct 26.3 in PRV-3 infected blood, and Ct 32.67 in the purified PRV-3 inoculum.

The finding of PRV-specific antibody response from 8 wpc directed against the outer capsid protein PRV-1 µ1C showed that the infection was recognized by the fish humoral response. Temporally, PRV-specific antibodies were detectable 2 weeks following the peak viral load and presumably the main shedding period which occurred at 6 wpc in cohabitants.

PRV-3 infection in rainbow trout did not cause reduced survival, neither in the positive control group nor in the group challenged with purified PRV-3 particles, consistent with what is previously observed under experimental conditions [12].

PRV-3 challenged fish developed typical heart pathology; a pancarditis, where all parts of the heart were affected. This was more pronounced in fish challenged with PRV-3 infected blood, nevertheless fish exposed to purified PRV-3 particles had these heart lesions. Although mild, the histopathological findings in the experimental group were consistent with a pathology described in field cases [11], from previous experiments [12] and the same as observed in the positive control group in this study.

An attempt to stain PRV antigen by immunohistochemistry in heart sections from fish with high viral loads with a polyclonal antibody against PRV-1 failed. This could be explained by low sensitivity of the antibodies in IHC, i.e. the amount of viral antigen was too low for detection. Another explanation could be conformational differences between PRV-1 and PRV-3. PRV-3 antigen can be detected in western blots under denaturing conditions, but it highlights the need for development of specific tools for identifying the localization of PRV-3 in tissue.

Our results linked the presence of the virus to the consecutive development of pathology in the heart, but furthermore also to the upregulation of CD8 and CD4 lymphocyte markers in this organ. The PRV-3 load peaked at 6 wpc in cohabitants, 4 weeks before the increased expression of CD8+ T cell population marker in the heart.

The increased level of the CD8+ T cell transcripts was associated with the observation of heart pathology, as previously observed in Atlantic salmon [32, 34]. This finding strengthens the link between CD8+ cytotoxic cells and heart inflammation, and indicates that the mechanisms of Norwegian PRV-1 induced HSMI [34] is paralleled by the pathogenesis of the PRV-3 induced heart pathology. The study highlighted some similarities between PRV-1 infection in Atlantic salmon and PRV-3 infection in rainbow trout. The two viral subtypes shares 80% homology at the genetic level [13], they target the respective host erythrocytes for replication and cause similar inflammatory lesion in the heart of infected fish. Both viral subtypes trigger similar immune responses in the respective hosts. Conversely, PRV-1 establishes a persistent infection in Atlantic salmon while our findings indicate that PRV-3 infection could be of limited duration in rainbow trout. Notably, PRV-3 affected rainbow trout in field outbreaks suffer severe anaemia [11], which is not reported for HSMI in Atlantic salmon. Furthermore typical PRV-3 outbreaks in rainbow trout occurred in fresh water, whereas HSMI is most common after sea transfer.

The innate antiviral gene regulation in the present study followed the PRV-3 kinetics, thus strengthening the association between PRV load and innate antiviral responses in infected salmonids [3, 12, 34, 35]. The innate antiviral gene upregulation in the early stage of PRV-3 infection may efficiently hamper the progression of viral infection, and lead to the decreased viral load after 6 wpc. Differences have been observed for HSMI and in innate antiviral response after PRV-1 challenge of Atlantic salmon indicating that viral and/or host genetic factors influence the outcome of a PRV-1 infection [3, 33]. For *Mammalian orthoreovirus* it has been shown that the ability to induce IFN contributes to the differences in the development of myocarditis [36, 37].

Previous studies [12] have shown how experimental infection in Atlantic salmon with PRV-3 infected blood failed to induce host innate immune response. Moreover, experimental infection in sockeye salmon (*Oncorhynchus nerka*) with PRV-1 fail to induce IFN related genes [38], whereas PRV-1 infection successfully trigger IFN related immune response modulating the susceptibility of Atlantic salmon to subsequent challenge with Infectious haematopoietic necrosis virus—IHNV or Salmonid alphavirus—SAV [35, 39, 40].

PRV infection of farmed salmonids can broadly be divided into two different clinical manifestations. (1) An acute disease, EIBS, which is a direct consequence of severe anemia at the peak of viremia, as seen in PRV-2 infection of coho salmon. It could be speculated that the jaundice syndrome described in Chinook salmon (related to PRV-1) [41] and jaundice syndrome reported in coho salmon (related to PRV-3) [16, 17] are downstream consequences of the clearance process of infected erythrocytes. Yet the role of PRV-1 and PRV-3 in the development of anaemia remains unknown. PRV-1 infection under experimental conditions failed to induce anaemia in Sockeye salmon [33, 38]. In Atlantic salmon, experimental infection with PRV-1 has only occasionally led to reduction of haematocrit and haemoglobin levels [35]. Notably, in this study, high loads of PRV-3 were generated in rainbow trout without causing a reduction of haematocrit, albeit it is described in disease outbreaks associated with PRV-3 in the field [11]. These findings might suggest that other factors, possibly related to farming conditions, are involved in the development of anaemia in clinical outbreaks. (2) HSMI, which appears a few weeks after the viraemia peak as demonstrated for PRV-1 in Atlantic salmon, is characterized by inflammation seen as influx of CD8 lymphocytes in heart tissue [34, 42]. In field outbreaks associated with PRV-3 reported by Olsen et al. [11], 113 fish were examined for histopathology. Amongst these, 80 fish showed evident clinical signs and 33 apparently looked healthy. A heart pathology resembling HSMI was described in 103 of the 113 fish examined [11]. In the current study, no clinical signs were observed, and an HSMI-like pathology was observed in a limited number of fish. Yet, in line with the timing of observation of heart pathology after PRV-1 infection in Atlantic salmon [19], the HSMI related histopathological findings in the heart in the present experiment were observed in the later stage of the trial, i.e. at 6 wpc in the injected group and 10 wpc in the cohabitant group.

In conclusion, the findings of this study support the hypothesis that establishes the causative relationship between PRV-3 infection in rainbow trout and the development of pancarditis as described for HSMI in Atlantic salmon. Furthermore, the identification of the aetiological agent, makes a basis for the development of specific preventive tools and control strategies. The recent emergence of PRV-3 variants in rainbow trout farms associated with severe disease outbreaks [13], warrants further investigation in search of factors that modulate the severity of the disease.

## Additional file

**Additional file 1.** **Proportion of fish showing histopathological findings not consistent with PRV-induced inflammation.** The findings consisted of epicarditis and a focal inflammatory reaction involving the interface layer between *stratum compactum* and *stratum spongiosum* of the ventricle. These were randomly distributed throughout the experiment and observed in all groups.
