# Supplementary material for: Piscine orthoreovirus subtype 3 (PRV-3) causes heart inflammation in rainbow trout (Oncorhynchus mykiss)
Source: Vet Res. 2019 Feb 18;50:14. doi: 10.1186/s13567-019-0632-4 (PMC6380033; doi:10.1186/s13567-019-0632-4)
Supplement: Supplementary file 1 — Additional file 1. Proportion of fish showing histopathological findings not consistent with PRV-induced inflammation. The findings consisted of epicarditis and a focal inflammatory reaction involving the interface layer between stratum compactum and stratum spongiosum of the ventricle. These were randomly distributed throughout the experiment and observed in all groups. [file 13567_2019_632_MOESM1_ESM.docx]

|  | Neg. control | | Purified PRV-3 | | PRV-3 infected blood | |
| --- | --- | --- | --- | --- | --- | --- |
| WPC | Shedder | Cohab. | Shedder | Cohab. | Shedder | Cohab. |
| 2 |  |  |  |  |  |  |
| 4 | 1 / 2 |  |  |  | 2 / 6 |  |
| 6 |  | 2 / 2 |  |  |  |  |
| 8 | 1 / 2 |  | 2 / 6 | 2 / 6 | 1 / 6 |  |
| 10 |  |  | 1 / 6 | 4 / 6 | 1 / 6 | 1 / 6 |
